# Impact of Treatment Modalities on Survival of Patients With Locoregional Esophageal Squamous-Cell Carcinoma in Taiwan

**DOI:** 10.1097/MD.0000000000003018

**Published:** 2016-03-11

**Authors:** Hui-Shan Chen, Wei-Heng Hung, Jiunn-Liang Ko, Po-Kuei Hsu, Chia-Chuan Liu, Shiao-Chi Wu, Ching-Hsiung Lin, Bing-Yen Wang

**Affiliations:** From the Institute of Health and Welfare Policy, National Yang-Ming University, Taipei (HSC, SCW); Division of Thoracic Surgery, Department of Surgery, Changhua Christian Hospital (WHH, BYW); Institute of Medicine, Chung Shan Medical University, Taichung (JLK, BYW); Department of Medical Oncology and Chest Medicine, Chung Shan Medical University Hospital (JLK); Division of Thoracic Surgery, Department of Surgery, Taipei Veterans General Hospital and National Yang-Ming University School of Medicine (PKH); Division of Thoracic Surgery, Department of Surgery, Koo Foundation Sun Yat-Sen Cancer Center, Taipei (CCL), Division of Chest Medicine, Department of Internal Medicine, Changhua Christian Hospital, Changhua (CHL); Department of Respiratory Care, College of Health Sciences, Chang Jung Christian University, Tainan (CHL); School of Medicine, Kaohsiung Medical University, Kaohsiung (BYW); and Institute of Genomics and Bioinformatics, National Chung Hsing University, Taichung, Taiwan (BYW).

## Abstract

The optimal treatment modality for locoregional esophageal squamous-cell carcinoma (ESCC) is still undetermined. This study investigated the treatment modalities affecting survival of patients with ESCC in Taiwan.

Data on 6202 patients who underwent treatment for locoregional esophageal squamous-cell carcinoma during 2008 to 2012 in Taiwan were collected from the Taiwan Cancer Registry. Patients were stratified by clinical stage. The major treatment approaches included definitive chemoradiotherapy, preoperative chemoradiation followed by esophagectomy, esophagectomy followed by adjuvant therapy, and esophagectomy alone. The impact of different treatment modalities on overall survival was analyzed.

The majority of patients had stage III disease (n = 4091; 65.96%), followed by stage II (n = 1582, 25.51%) and stage I cancer (n = 529, 8.53%). The 3-year overall survival rates were 60.65% for patients with stage I disease, 36.21% for those with stage II cancer, and 21.39% for patients with stage III carcinoma. Surgery alone was associated with significantly better overall survival than the other treatment modalities for patients with stage I disease (*P* = 0.029) and was associated with significantly worse overall survival for patients with stage III cancer (*P* < 0.001). There was no survival risk difference among the different treatment methods for patients with clinical stage II disease.

Multimodality treatment is recommended for patients with stage II–III esophageal squamous-cell carcinoma. Patients with clinical stage I disease can be treated with esophagectomy without preoperative therapy.

## INTRODUCTION

Esophageal squamous-cell carcinoma (ESCC) is a very aggressive cancer and is one of the leading causes of cancer-related death. The disease is associated with dismal survival rates, with the majority of patients dying within 1 year of diagnosis.^1–3^ In Taiwan, squamous-cell carcinoma is the most common type of esophageal cancer and most patients have advanced stage disease when it is 1st diagnosed.^2^ The long-term prognosis of ESCC remains poor regardless of the treatment approach.

The National Comprehensive Cancer Network (NCCN) guidelines for esophageal cancer^4^ recommend that patients with stage T1N0 ESCC receive endoscopic therapies or esophagectomy and that patients with clinical stage T2 (or higher) or node-positive disease should undergo definitive chemoradiation therapy (CRT), CRT followed by esophagectomy, or esophagectomy alone. Postoperative CRT is recommended after R1/R2 resection. Ideally, the treatment strategy should be tailored to the patient's performance status and preference. Combination therapies such as chemotherapy, radiation, and esophagectomy may result in better survival in patients with locoregional ESCC. However, the optimal treatment combination and sequence for locally advanced ESCC has yet to be determined.

The purpose of this study was to investigate the influence of different treatment modalities on overall survival of patients who were treated for locoregional ESCC throughout Taiwan during the period 2008 to 2012.

## MATERIALS AND METHODS

Patient data were obtained from the Taiwan Cancer Registry, an annually updated national population database. The database contains information on age at diagnosis, sex, cancer type, care facilities, clinical stage, pathologic stage, surgical margin status, tumor location, tumor grade, and treatment modality. Causes of death are linked to the National Register of Deaths Database. Since these were deidentified secondary data, released for public access for research purposes, the study was exempt from full review by the Internal Review Board in our hospital. In this study, we searched the Taiwan Cancer Registry for all patients who were treated for locoregional ESCC during the period 2008 to 2012. The following International Classification of Disease for Oncology site codes were used to identify eligible patients: C15.0, C15.1, C15.2, C15.3, C15.4, C15.5, C15.8, and C15.9 as well as codes 8052, 8070, 8071, 8072, 8073, 8074, 8076, 8077, 8083, and 8044. A total of 9407 patients with ESCC were identified. Of those patients, 90 (1%) had stage 0 disease, 589 (6%) had stage I disease, 1678 (17%) received treatment for stage II cancer, 4329 (44%) had stage III disease, and 2721 (27%) had stage IV cancer. Since the aim of this study was to investigate the impact of treatment modalities on survival of patients with locoregional ESCC, we excluded patients with either stage 0 or stage IV disease. Therefore, 6596 patients were selected.

The 6596 selected patients underwent one of the following primary treatment modalities: definitive CRT (n = 2848, 43.2%); neoadjuvant CRT followed by esophagectomy (n = 1459, 17.6%); esophagectomy alone (n = 831, 12.6%); esophagectomy followed by chemotherapy or radiotherapy or both (n = 628, 9.5%); radiotherapy alone (n = 439, 6.7%); chemotherapy alone (n = 241, 3.7%); other types of treatment (n = 52, 0.8%); or unknown types of treatment (n = 394, 6.0%). Of the 628 patients receiving esophagectomy followed by adjuvant therapy, 115 patients underwent adjuvant chemotherapy, 406 patients underwent adjuvant chemoradiotherapy, and 107 patients underwent adjuvant radiotherapy. Patients with unknown treatment (n = 394) were excluded from the analysis. Therefore, a total of 6202 patients were included in this study.

The clinical and histologic factors included in the analysis were age, sex, clinical T stage, clinical N stage, pathologic stage, surgical margin status, tumor location, histologic grade, treatment modality, and 1-, 2-, and 3-year survival rates. Patients were stratified into 1 of 5 major treatment groups depending on the treatment they received during the study period, namely definitive CRT (n = 2848, 45.92%), preoperative CRT followed by esophagectomy (n = 1163, 18.75%), esophagectomy alone (n = 831, 13.4%), esophagectomy followed by chemotherapy or radiotherapy or both (n = 628, 10.13%), and other alternative treatments (n = 732, 11.80%).

All tumor specimens were graded histologically based on the World Health Organization classification of esophageal cancers and were staged according to the tumor-node-metastasis staging system (American Joint Committee on Cancer, cancer staging manual, 7th edition).^5^

## STATISTICAL ANALYSIS

Overall survival was measured from the date of initial treatment for esophageal cancer to the date of death due to any cause or the censoring date of December 31, 2013. The causes and dates of death were obtained from the National Register of Deaths Database (http://www.mohw.gov.tw/). Survival analysis was carried out using the Kaplan–Meier method and between-group differences in survival were determined by the log-rank test. We used the Chi-square test or Fisher's exact test for categorical comparisons of data. Differences in means of continuous variables were tested by the Student's *t*-test. Significant variables in the univariate analyses were then included in a stepwise multiple logistic-regression model to identify the most important predictors of survival for each clinical stage. Cox proportional-hazards analysis was used to determine the relative contribution of various factors to overall survival. A *P* value of <0.05 was considered to indicate statistical significance; all tests were 2-tailed. All statistical analyses were performed with the statistical software package SPSS (Version 17.0, SPSS Inc., Chicago, IL).

## RESULTS

Basic clinical data of the 6202 patients with clinical stage I–III are summarized in Table 1. The majority of patients had stage III disease (n = 4091; 65.96%), followed by stage II (n = 1582, 25.51%) and stage I cancer (n = 529, 8.53%). Nearly 95% of the patients were men (n = 5840, 94.16%). Of the 6202 patients, 2848 patients (45.92%) underwent definitive CRT, 1163 patients (18.75%) underwent preoperative CRT followed by esophagectomy, 628 patients (10.13%) underwent esophagectomy followed by adjuvant therapy, 831 patients (13.4%) underwent esophagectomy alone, and 732 patients (11.80%) underwent other treatments (such as chemotherapy only or radiation only). Data on pathologic stage and surgical margin status were only available for patients who underwent surgery.

**TABLE 1:**
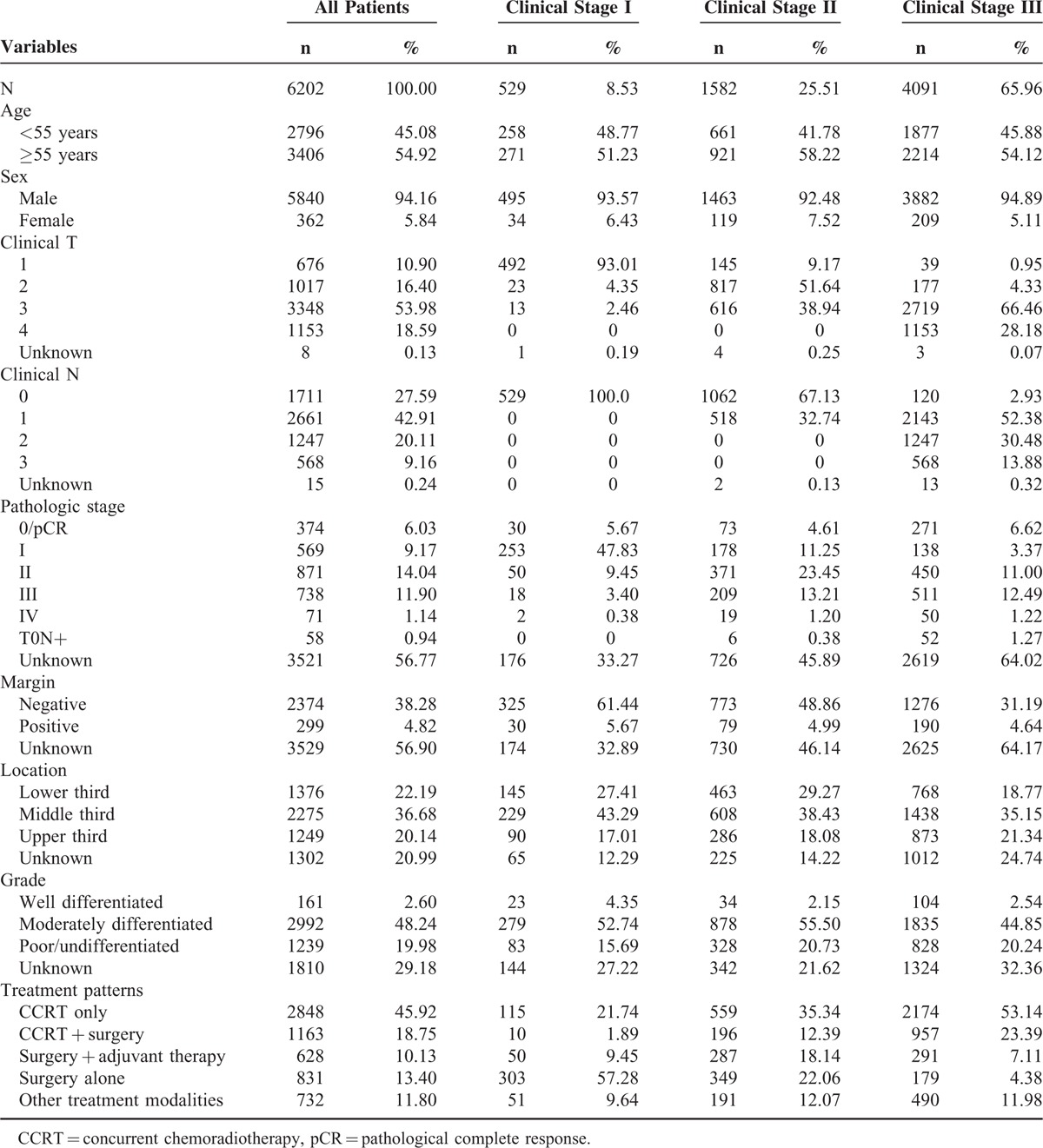
Clinical Data of 6202 Patients With Clinical Stage I–III Esophageal Squamous-Cell Carcinoma

**FIGURE 1:**
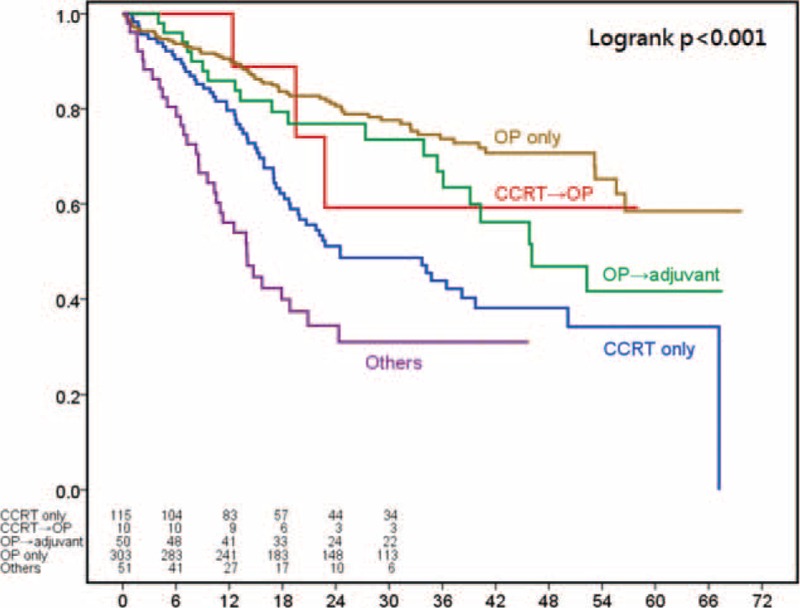
Kaplan–Meier survival curves for 529 patients with clinical stage I esophageal squamous-cell carcinoma stratified by treatment modalities.

Patients were stratified according to clinical stage to investigate the influence of treatment patterns on survival (Table 2). For patients with stage I disease, the most common treatment modality was surgery alone (n = 303) followed by definitive CRT (n = 115), other types of treatment (n = 51), surgery with adjuvant therapy (n = 50), and preoperative CRT followed by surgery (n = 10). The survival curves according to treatment patterns for patients with clinical stage I disease are shown in Figure 1. Surgery alone was associated with significantly better overall survival than the other treatment modalities (*P* < 0.001).

**TABLE 2:**
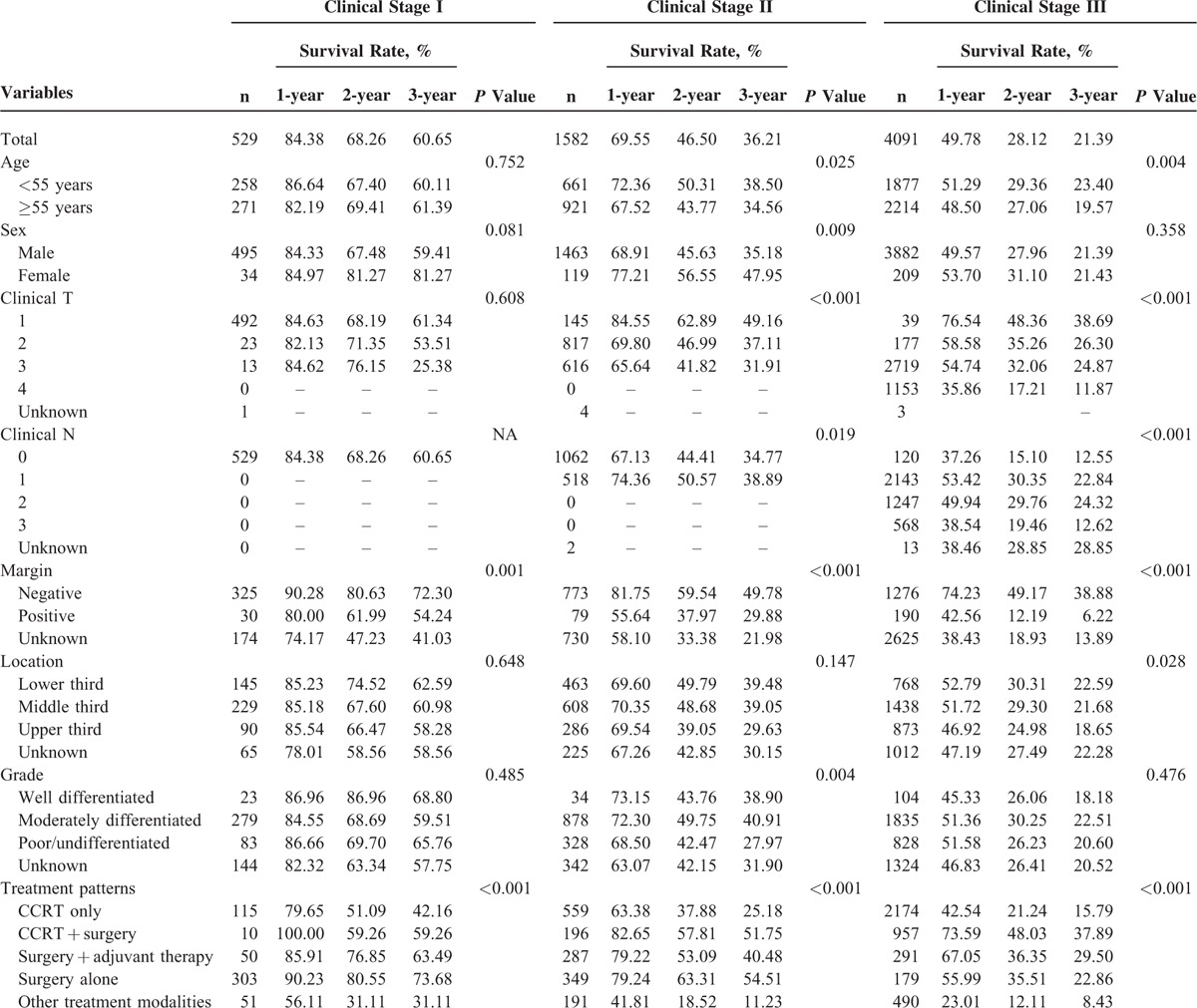
Univariate Analyses of Factors Associated With Overall Survival in Patients Stratified by Clinical Stage

**FIGURE 2:**
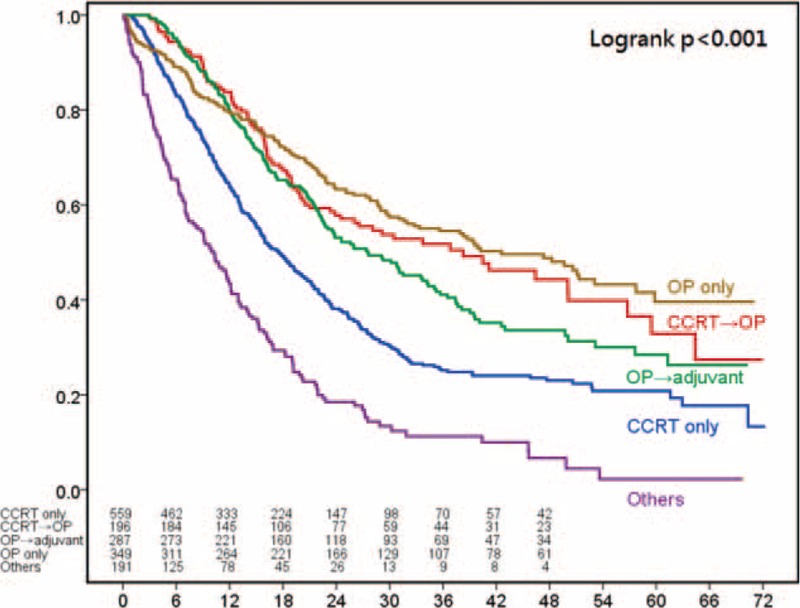
Kaplan–Meier survival curves for 1582 patients with clinical stage II esophageal squamous-cell carcinoma stratified by treatment modalities.

For patients with stage II disease, the most common treatment modality was definitive CRT (n = 559) followed by surgery alone (n = 349), surgery with adjuvant therapy (n = 287), preoperative CRT followed by surgery (n = 196), and other treatments (n = 191) (Table 2). The survival curves according to treatment patterns for patients with clinical stage II disease are shown in Figure 2. The 3-year survival rates were 25.18% for definitive chemoradiation, 51.75% for preoperative CRT followed by surgery, 40.48% for surgery plus adjuvant therapy, 54.51% for surgery alone, and 11.23% for other treatment types (*P* < 0.001).

**FIGURE 3:**
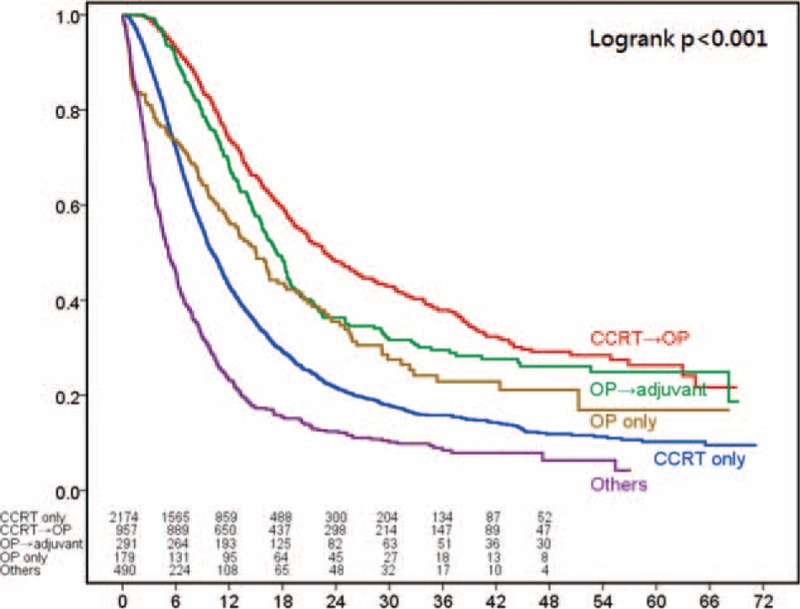
Kaplan–Meier survival curves for 4091 patients with clinical stage III esophageal squamous-cell carcinoma stratified by treatment modalities.

For patients with stage III disease, the most common treatment modality was definitive CRT (n = 2174) followed by preoperative CRT plus surgery (n = 957), surgery with adjuvant therapy (n = 291), and surgery alone (n = 179) (Table 2). The survival curves according to the treatment patterns are shown in Figure 3. Patients receiving preoperative CRT followed by surgery had significantly better 3-year survival rates than patients who received any of the other treatment patterns *P* < 0.001).

Significant variables in the univariate analyses (age, sex, surgical margin status, tumor location, histological grade, clinical T classification, clinical N classification, and treatment patterns, Table 2) were included in a multiple logistic-regression model to identify the most important factors associated with survival for each clinical stage (Table 3). Cox proportional-hazards analysis was then used to determine the relative contribution of the variables to overall survival. The hazard ratio was defined as 1 in patients who received definitive CRT. There was no survival risk difference among definitive CRT, preoperative CRT followed by surgery, and surgery with adjuvant therapy for patients with clinical stages I, II, and III. However, patients receiving alternative therapies had worse survival outcomes regardless of clinical stage. Surgery alone was associated with significantly better overall survival than definitive CRT for patients with stage I disease (hazard ratio = 0.28, *P* = 0.029) and was associated with significantly worse overall survival for patients with stage III cancer (hazard ratio = 1.78, *P* < 0.001).

**TABLE 3:**
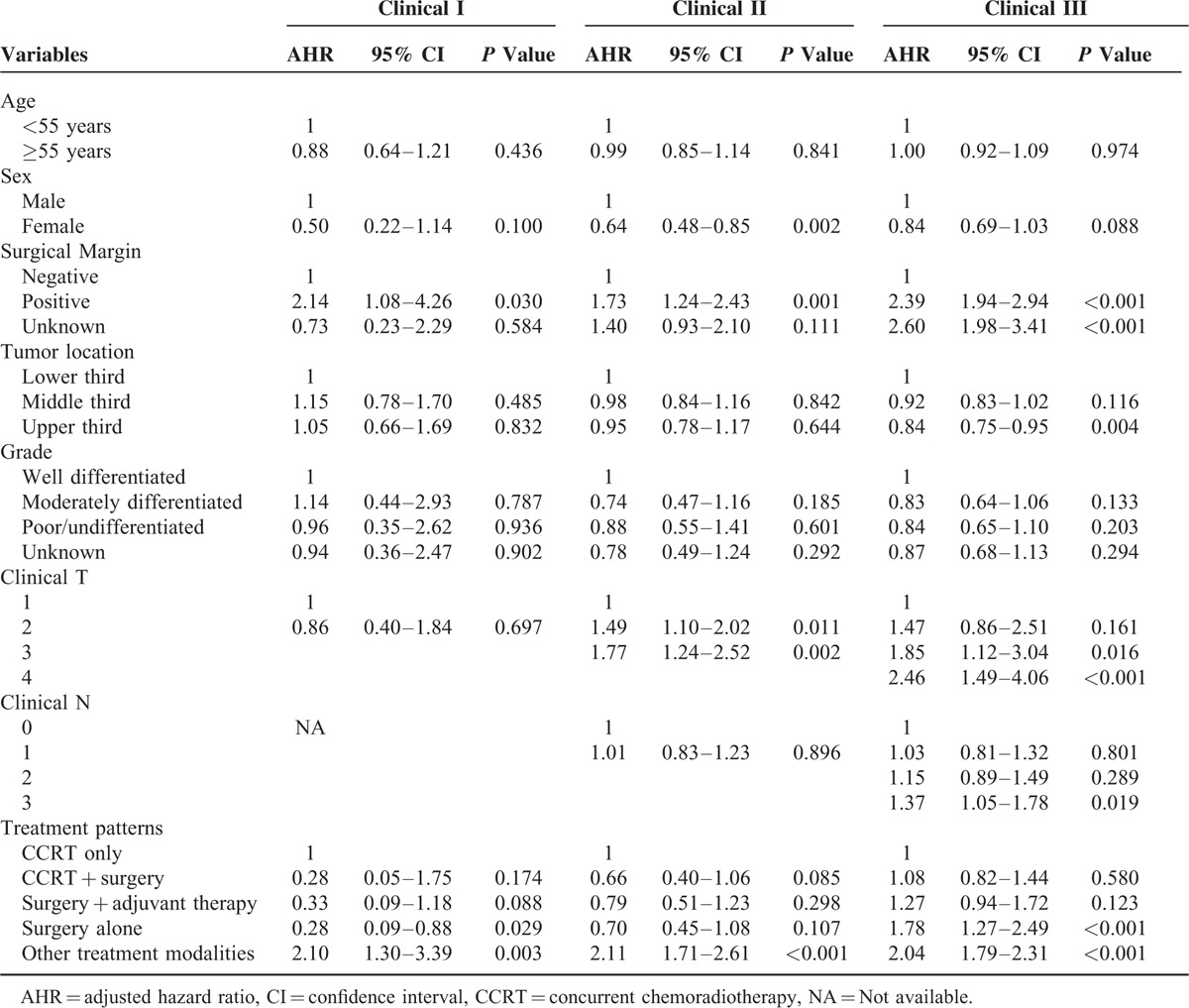
Multivariate Analyses for Overall Survival in Patients Stratified by Clinical Stage

In multivariate analysis (Table 3), surgical margin and treatment modality were the independent prognostic factors in patients with clinical stage I ESCC. Gender, surgical margin, clinical T, and treatment modality were the independent prognostic factors in clinical stage II patients. Regarding to clinical stage III patients, surgical margin, tumor location, clinical T, clinical N, and treatment modality were the independent prognostic factors for overall survival.

## DISCUSSION

In this study, we investigated the impact of different treatment modalities on overall survival of patients with locoregional ESCC in Taiwan. We found that esophagectomy alone was associated with better overall survival than definitive CRT in patients with clinical stage I disease (*P* = 0.029), that treatment modality was not associated with survival in patients with stage II cancer, and that esophagectomy alone was associated with worse survival than definitive CRT in patients with stage III ESCC (*P* < 0.001). Alternative treatments such as chemotherapy alone and radiation therapy alone were associated with poorer overall survival than definitive CRT regardless of ESCC stage (*P* = 0.003 for stage I; *P* < 0.001 for stage II; and *P* < 0.001 for stage III).

Definitive CRT is recommended by NCCN guidelines for most patients with advanced locoregional ESCC.^4^ The RTOG 85-01/INT123 trials established the protocol of definitive CRT (5-FU, cisplatin X4 plus concurrent 50 Gy) and described the therapeutic long-term survival.^6,7^ A number of retrospective cohort studies have shown that the rates of survival of patients with esophageal cancer who undergo definitive CRT are similar to those in patients who receive surgical treatment approaches.^8–12^ Motoori et al^11^ enrolled 173 patients with clinical T1bN0M0 squamous cell carcinoma of thoracic esophagus. In the study, 102 patients were treated with esophagectomy and 71 patients with definitive CRT. There was no statistically significant overall survival difference between 2 groups. Karran et al^12^ retrospectively studied 521 patients with clinical stage I–IVa esophageal cancer (277 in the surgery and 244 in the definitive CRT). They performed propensity score analysis to eliminate the selection bias and found there was no difference in survival after esophageal cancer treatment involving surgery or definitive CRT. Several randomized clinical trials have compared the effect of definitive CRT with that of other treatment modalities on overall survival of patients with esophageal cancer. For example, Bedenne et al^13^ studied 259 patients (88.8% ESCC) with operable T3N0-1 cancer and found that there was no significant difference in survival between patients who underwent surgery after CRT and patients who received definitive CRT. Stahl et al^14^ randomized 172 patients (100% ESCC) with locally advanced disease (T3-4N0-1) to receive either definitive CRT or CRT plus surgery and found that CRT followed by surgery did not improve survival. Teoh et al^15^ randomized 81 patients with resectable ESCC to receive either definitive CRT or esophagectomy alone and found that both treatment modalities resulted in comparable long-term survival rates. Other clinical trials have shown similar findings.^16–18^ In addition, a recent meta-analysis revealed that overall survival was equivalent between surgery and definitive CRT.^19^ The above-mentioned studies provide convincing evidence that CRT should be considered definitive treatment for locoregional esophageal cancer. In our study, we also excluded patients with stage IV disease to compare the different treatment modalities on overall survival of patients with locoregional ESCC. Patients were stratified according to clinical stage. We found that esophagectomy alone was associated with better overall survival than definitive CRT in patients with clinical stage I disease. Definitive CRT in patients with stage II and stage III ESCC was associated with similar survival compared with other treatment modalities

Studies have shown that definitive CRT results in complete pathologic response in 25% to 40.4% of cases,^20–25^ which implies that CRT alone is sufficient for select patients with esophageal cancer. Esophagectomy with lymph node dissection is a complex surgery and is associated with moderate postoperative mortality and morbidity.^20,22,26–28^ Therefore, the potential survival benefit of surgery is counterbalanced by its surgical risk. In Taiwan, almost half of patients (n = 2848, 45.9%) with locoregional ESCC received definitive CRT during the period 2008 to 2012. We found that for patients with stage I disease esophagectomy alone resulted in better overall survival than definitive CRT (*P* = 0.029). However, for patients with stage III ESCC, surgery alone was associated with significantly poorer overall survival than other treatments. For patients with stage II disease, there was no significant difference in overall survival among the major different treatment modalities. Therefore, definitive CRT could be a valuable treatment for patients with stage II or stage III locoregional esophageal squamous-cell carcinoma.

Multiple randomized clinical trials have compared the clinical impact of preoperative CRT followed by surgery with that of surgery alone and found that CRT plus esophagectomy is associated with better overall survival than surgery alone.^29–37^ Several meta-analyses reached a similar conclusion.^38–40^ However, 2 recent clinical trials provided inconsistent results. The CROSS trial, which enrolled 366 patients (cT1 1%, cT2 15%, cT3 84%, cT0 33%, cN1 65%, and 75% adenocarcinoma), concluded that preoperative CRT improved survival among patients with potentially curable esophageal cancer.^20^ In contrast, the FFCD 9901 trials, which included 195 patients with clinical I/II esophageal cancer (clinical I 19%, clinical IIA 53.3, clinical IIB 27.7%, and 90% squamous cell carcinoma), concluded that preoperative CRT not only does not improve survival compared with surgery alone, but also in fact is associated with increased postoperative surgical mortality in patients with stage I or stage II disease.^22^ In our study, we found that the 3-year overall survival rate was similar between patients with clinical stage I or II disease who were treated with CRT plus surgery and those who received surgery alone (Table 2). However, 3-year overall survival was markedly worse in patients with stage III ESCC who received esophagectomy than in those who received CRT prior to esophagectomy (*P* < 0.001) (Table 2). Complete surgical resection with lymph node dissection may increase the chance of long-term survival. Based on our analysis, patients with clinical stage III ESCC should undergo multimodality treatment rather than esophagectomy alone.

The INT-0116 trials showed that postoperative CRT is beneficial for patients with adenocarcinoma of the stomach or gastroesophageal junction.^41,42^ Two clinical trials failed to demonstrate survival benefit of adjuvant therapy for patients receiving esophagectomy.^43,44^ Several retrospective studies have shown that adjuvant therapy is associated with better survival.^23,24,45–50^ The benefit of adjuvant therapy for esophageal cancer was still undetermined. Postoperative adjuvant therapy is not recommended for patients with ESCC according to current NCCN practice guidelines unless the surgical margins are positive.^4^ In our study, there were no significant differences in overall survival between patients with stage I–II disease who received esophagectomy with adjuvant therapy and patients who received surgery alone. In the multivariate analysis, surgery without adjuvant therapy was an independent predictor of adverse outcome (*P* < 0.001). Patients with clinical stage III ESCC, therefore, might benefit from adjuvant therapy.

Complete surgical resection of esophageal cancer could provide curative chance. Javidfar et al^51^ enrolled 3125 patients with pathologic T1-3N0-1M0 esophageal cancer from 2003 to 2006 in the National Cancer Data Base into analysis and found positive surgical margin are associated with poor survival. A systemic review^52^ identified positive margin to be also associated with overall poor survival. We also concluded that positive surgical margin was an independent predictor of poor prognosis by multivariable analysis in stage I–II–III ESCC patients.

The strength of our study was its large population size. We used a large population database to identify patients with locoregional ESCC to investigate the efficacy of definitive CRT. There were several limitations in the study, including its retrospective nature and the heterogeneity of chemotherapy regimen data and radiation dose. Information on treatment rationale and patient preference is not available in the database. Therefore, the results should be interpreted with caution.

## CONCLUSION

Based on our analysis of 6202 patients with ESCC in Taiwan who underwent different treatments, we found that treatment modality was associated with overall survival. Thoughtful multidisciplinary deliberations are the cornerstone for determining the optimal multimodality strategy best suited to each patient.
